# Comparison of DL-Methionine and L-Methionine levels in liver metabolism activity in commercial broilers fed a diet without antibiotic growth promoters

**DOI:** 10.14202/vetworld.2025.598-605

**Published:** 2025-03-18

**Authors:** Andriyanto Andriyanto, Tiok Bagus Taufani Sanoesi, Aditya Ananda Putra, Mawar Subangkit, Amaq Fadholly, Hamdika Yendri Putra, Dordia Anindita Rotinsulu

**Affiliations:** 1 Division of Pharmacology and Toxicology, School of Veterinary Medicine and Biomedical Sciences, IPB University, West Java16680, Indonesia; 2 PT. Cheil Jedang Indonesia, South Jakarta, Jakarta 12710, Indonesia; 3 Division of Pathology, School of Veterinary Medicine and Biomedical Sciences, IPB University, West Java16680, Indonesia; 4 eLRosa Laboratory, iRATco Group, 16680 West Java, Indonesia; 5 Division of Medical Microbiology, School of Veterinary Medicine and Biomedical Sciences, IPB University, West Java16680, Indonesia

**Keywords:** antioxidant activity, broiler chickens, DL-Methionine, liver metabolism, L-Methionine

## Abstract

**Background and Aim:**

Methionine is an essential amino acid in broiler nutrition, playing a crucial role in growth performance and liver metabolism. As an alternative to antibiotic growth promoters (AGPs), this study aimed to evaluate the effects of DL-Methionine and L-Methionine supplementation on liver metabolism, antioxidant activity, and growth performance in broilers raised without AGPs.

**Materials and Methods:**

A total of 440 one-day-old Cobb 500 male broiler chicks were assigned to 11 groups: A control group and 10 treatment groups receiving graded levels (70%, 85%, 100%, 115%, and 130%) of either DL-Methionine or L-Methionine. The study assessed body weight, liver mass index, D-amino acid oxidase (DAAO) activity, serum glutathione concentration, and liver histopathology across three growth stages: Starter (day 11), grower (day 25), and finisher (day 35).

**Results:**

L-Methionine supplementation resulted in greater body weight gain compared to DL-Methionine, particularly in the finisher stage. DL-Methionine demonstrated a stronger influence on liver metabolism by increasing DAAO activity and reducing oxidative stress, as indicated by lower serum glutathione levels. No significant histopathological alterations were observed among the groups, confirming the safety of both methionine sources.

**Conclusion:**

While L-Methionine improved growth performance, DL-Methionine effectively enhanced liver metabolism and reduced oxidative stress. These findings suggest that DL-Methionine may support liver function, whereas L-Methionine is more effective for weight gain in broilers raised without AGPs.

## INTRODUCTION

Antibiotic growth promoters (AGPs) have long been used in broiler production to enhance growth performance and feed efficiency. Their mechanisms of action include modulation of the microbiota, immune regulation, reduction of inflammation, and metabolic optimization. By altering the composition of the gut microbiota, AGPs selectively suppress pathogenic bacteria while promoting beneficial micro-organisms, thereby improving nutrient absorption and use. This effect enhances growth performance [1]. However, the widespread use of AGPs has raised concerns about antibiotic resistance and the potential transfer of resistant bacteria or residues to humans through poultry products. These concerns have prompted the search for alternatives, such as optimizing feed formulations to meet broiler chickens’ nutritional needs. Essential amino acids, such as methionine, have emerged as a viable replacement strategy for AGPs without compromising growth performance.

Methionine is an essential amino acid that must be supplied through the diet because broiler chickens cannot synthesize it endogenously. It plays a crucial role in protein synthesis, feather development, immune function, and metabolism. Methionine also serves as a precursor for cysteine and taurine and is involved in the synthesis of proteins and enzymes essential for growth and overall health. Adjusting the dietary ratio of methionine, cysteine, and lysine significantly influences growth performance, carcass composition, and feather coverage in broilers. Methionine supplementation has also been shown to strengthen the immune system, enhance disease resistance, reduce antibiotic reliance, and improve carcass quality. In contrast, insufficient dietary methionine negatively affects growth rates, feed efficiency, and overall carcass yield [2, 3].

In liver metabolism, methionine is a pivotal precursor for various biochemical pathways. It is the first amino acid incorporated into nascent polypeptide chains during protein synthesis and is converted into S-adenosylmethionine (SAMe), a key methyl donor involved in DNA, RNA, protein, and lipid methylation [4, 5]. In addition, methionine participates in the trans-sulfuration pathway, leading to cysteine synthesis, which supports glutathione production, a critical antioxidant in liver metabolism.

DL-Methionine and L-Methionine are commonly used in broiler feed formulations. DL-Methionine is a racemic mixture of D- and L-isomers, whereas L-Methionine is the biologically active form involved in protein synthesis and metabolic pathways [6]. Previous studies reported by Bohatko [7], Son *et al*. [8], Yu *et al*. [9] suggest that L-Methionine is metabolized and utilized more efficiently than DL-Methionine. L-Methionine supports higher incorporation into proteins and SAMe production, whereas DL-Methionine may undergo alternative metabolic pathways, potentially accumulating toxic intermediates such as homocysteine. Ensuring adequate L-Methionine levels in the diet is essential for maintaining optimal liver function, growth, and overall health in broilers [10]. This study focuses on evaluating the efficiency of DL-Methionine and L-Methionine in supporting liver metabolism in commercial broiler chickens raised without AGP supplementation. By comparing their metabolic and physiological impacts, this study determines the optimal methionine source for enhancing broiler health and performance in AGP-free production systems.

## MATERIALS AND METHODS

### Ethical approval

This study was approved by the Animal Ethics Committee, School of Veterinary Medicine and Biomedical Sciences, IPB University, Bogor, Indonesia (No.100/KEH/SKE/VIII/2023).

### Study period and location

This study was conducted from October 2023 to December 2023 at the Laboratory of Animal Management Unit, School of Veterinary Medicine and Biomedical Sciences, IPB University.

### Experimental animals

This study used 440 one-day-old (DOC) Cobb 500 male broiler chicks from PT. CJ Indonesia. No acclimatization was performed on the experimental animals because treatment had to be administered from the 1^st^ day of birth. The chickens were divided into 11 groups: A control group (no treatment) and two treatment groups (P0), each receiving five different concentrations of P1 (70%), P2 (85%), P3 (100%), P4 (115%), and P5 (130%) of DL-Methionine and P6 (70%), P7 (85%), P8 (100%), P9 (115%), and P10 (130%) of L-Methionine. Each group contained 40 animals and was placed in a cage with dimensions of 4 m in length, 3 m in width, and 1.5 m in height. The research animals were kept in an intensive closed cage system with standard rearing practices and were completely vaccinated. Feed and water are provided *ad libitum*. The chickens were euthanized by considering animal welfare principles through exsanguination at 3-time points: Day 11 (starter stage), day 25 (grower stage), and day 36 (finisher stage). At each stage of age, the chickens weresacrificed with exsanguination methods for data collection. In the starter and grower stages, 15 animals were sacrificed, and in the finisher stage, 14 animals were sacrificed.

### Body weight and liver mass index (LMI)

The body weight and LMI of broilers were measured to evaluate growth performance and liver health. The LMI was calculated by dividing the liver weight by the body weight and multiplying by 100 to obtain the percentage, which reflects liver development and potential health issues. Regular monitoring of body weight and LMI is essential for assessing the effectiveness of dietary interventions and management practices for broiler production to ensure optimal growth and well-being.

### D-amino acid oxidase (DAAO) concentration

The DAAO test on liver samples involves several steps. The liver tissue samples were first homogenized and centrifuged to isolate the supernatant, which contained the enzymes. A reaction mixture was then prepared using the liver supernatant, a substrate (usually a D-amino acid such as D-alanine or D-serine), and a cofactor (flavin adenine dinucleotide). After concentrating the mixture and stopping the reaction, the resulting hydrogen peroxide was measured using spectrophotometry or specific detection reagents. DAAO activity was calculated based on the amount of product formed, typically expressed in units per milligram of protein or per gram of tissue.

### Serum glutathione concentration

Serum samples were collected and stored at appropriate temperatures to prevent degradation. The glutathione concentration was measured using an enzyme-linked immunosorbent assay kit following the manufacturer’s instructions. The serum samples were diluted in the assay buffer to the recommended concentration. Microtiter plate wells were coated with glutathione capture antibodies and blocked to minimize non-specific binding. Diluted serum samples and glutathione standards were added to the wells and incubated to allow glutathione to bind to the capture antibody. After washing to remove unbound substances, a detection antibody specific to glutathione was added, followed by incubation and washing. Then, a secondary enzyme-conjugated antibody, which binds to the detection antibody, was added. After the final washing step, the substrate solution was introduced, and the reaction produced a color change proportional to the amount of glutathione bound. The absorbance was measured at specific wavelengths using a microplate reader. Glutathione concentrations were determined by comparing absorbance values with a standard curve generated from known glutathione concentrations.

### Liver histopathology score

Liver tissue samples were processed using standard histological techniques, including dehydration, cleaning, and embedding in paraffin wax. Thin-embedded tissue sections were cut using a microtome and mounted on glass slides. The sections were stained with hematoxylin and eosin to visualize the cellular structures and tissue architecture. A histopathological scoring system was applied to assess liver damage, evaluating parameters such as hepatocellular degeneration, necrosis, inflammation, fibrosis, and lipid accumulation. The individual scores for each parameter were then summed to calculate the total histopathological score for each liver sample to provide an overall assessment of liver toxicity.

### Statistical analysis

The statistical analysis was conducted using GraphPad Prism (version 6.0.1, GraphPad Software, Inc., USA). Data were tested for normality using the Shapiro–Wilk test and for homogeneity of variance using Levene’s test. Normally distributed data were analyzed using one-way analysis of variance (ANOVA) to compare differences among treatment groups, followed by Tukey’s honest significant difference test for *post ho*c multiple comparisons. For comparisons involving only two groups, an independent t-test was used. Repeated measures ANOVA was applied to evaluate changes across different growth stages (starter, grower, and finisher), with Bonferroni correction for *post ho*c pairwise comparisons. Pearson’s correlation analysis was performed to assess the relationships between liver metabolism markers, including DAAO activity and serum glutathione concentration, and growth performance indicators such as body weight and liver mass index. If data did not meet normality or homogeneity assumptions, non-parametric tests were used, including the Kruskal–Wallis test as an alternative to ANOVA and the Mann–Whitney U-test in place of the t-test. Categorical data from histopathological analysis were compared using the Chi-square test or Fisher’s exact test. The significance threshold was set at p < 0.05 for all analyses.

## RESULTS

### Body weight and LMI

The body weight assessment revealed that during the starter stage (day 11), there was no significant increase in body weight among all groups, including the control group. In the grower stage (day 25), the highest mean body weight was observed in the P9 group, followed by the P5 group. However, statistically, no significant differences were observed among the groups. By the finisher stage (day 35), the groups starting from P5 to P8 demonstrated significantly higher body weight increases compared with the other groups (Table 1).

**Table 1:** Comparison of body weight changes across three distinct ages: Day 11 (starter stage), day 25 (grower stage), and day 35 (finisher stage).

Group	Days (grams)		

	11	25	35
P0	384.0 ± 45.5^ns^	548.6 ± 76.9^ns^	1733.5 ± 168.4^bc^
P1	379.6 ± 37.3^ns^	556.8 ± 92.6^ns^	1600.3 ± 148.7^b^
P2	379.2 ± 48.6^ns^	605.2 ± 95.3^ns^	1725.2 ± 105.8^bc^
P3	377.1 ± 45.3^ns^	630.4 ± 123.5^ns^	1785.7 ± 148.2^bc^
P4	364.7 ± 35.4^ns^	500.6 ± 103.9^ns^	1794.2 ± 167.8^bc^
P5	368.6 ± 25.3^ns^	657.6 ± 137.1^ns^	1843.0 ± 158.0^bc^
P6	350.4 ± 85.6^ns^	653.6 ± 156.7^ns^	1856.9 ± 174.6^cd^
P7	398.6 ± 32.9^ns^	613.2 ± 170.8^ns^	1895.9 ± 156.1^cd^
P8	401.5 ± 24.4^ns^	621.7 ± 141.1^ns^	1934.2 ± 165.3^d^
P9	385.4 ± 16.3^ns^	666.6 ± 139.6^ns^	1367.4 ± 296.6^a^
P10	368.1 ± 24.8^ns^	521.9 ± 176.8^ns^	1186.0 ± 117.9^a^

ns refers to the no significant difference between the data points compared to P0. The superscript letter in the same column refers to the significant difference between each data in p ≤ 0.05

**Figure 1:**
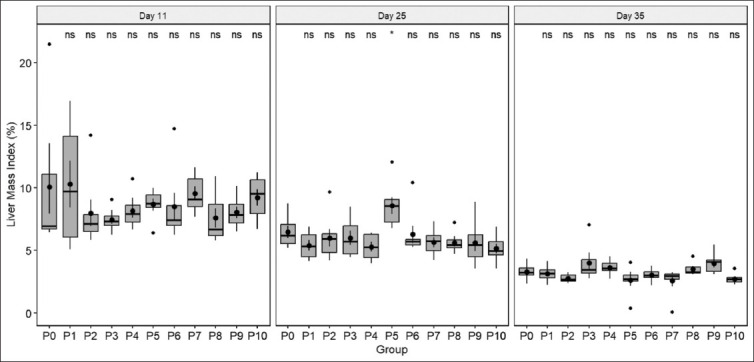
Comparison of liver mass index changes across three stages: Day 11 (starter stage), day 25 (grower stage), and day 35 (finisher stage). The dot inside each bar represents the mean value, and the stripes inside each bar represent the median value. The dots outside the bar indicate outliers. ns refers to no significant difference between each data point compared to P0, while (*) imply a significant value with p < 0.05.

During the starter and grower stages, no significant differences in LMI were observed between the treatment and control groups. However, in the starter group, most L-Methionine groups exhibited a more extensive mean LMI value compared with L-Methionine groups. In the finisher stage, the L-Methionine group had a lower LMI than the DL-Methionine group (Figure 1).

### DAAO concentration

**Figure 2:**
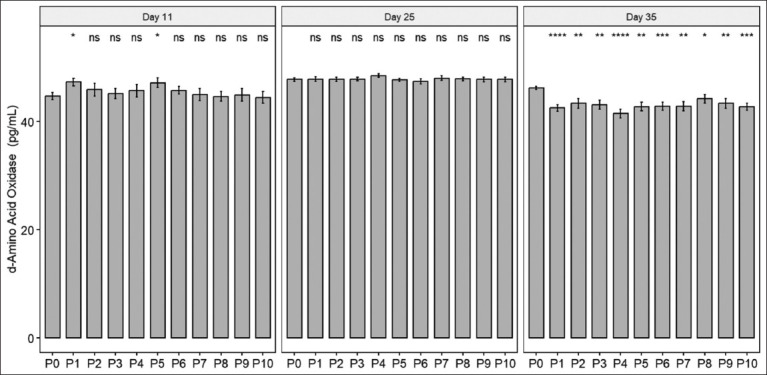
Comparison of D-amino acid oxidase concentrations in livers across three stages: day 11 (starter stage), day 25 (grower stage), and day 35 (finisher stage). The stripes at the top of each bar represent the standard deviation.

In the starter group, compared with P0, both DL-Methionine in P1 and P4 demonstrated higher concentrations of DAAO than P0. The group given L-Methionine had no significant difference from P0. During the grower stage, no significant differences were observed between the groups. However, by the finisher stage, it was obvious that all treatment groups, both DL-Methionine and L-Methionine groups, showed lower concentrations of DAAO compared to the control group (Figure 2).

### Serum glutathione concentration

**Figure 3:**
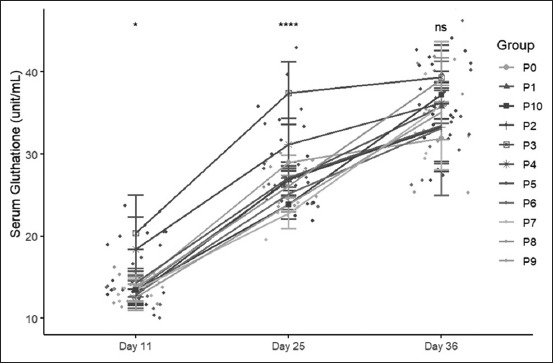
Comparison of glutathione concentration in serum changes across three stages: Day 11 (starter stage), day 25 (grower stage), and day 35 (finisher stage).

At the starter stage, the serum glutathione concentrations in the treatment and control groups differed significantly, with the P3 group showing the highest mean value compared with the other groups. At the grower stage, all groups showed an increase in serum glutathione levels, and the mean serum glutathione level in the P3 group remained higher than that in the other groups. All groups did not exhibit significant differences at the finisher stage, as shown in Figure 3.

### Liver histopathology score

**Figure 4:**
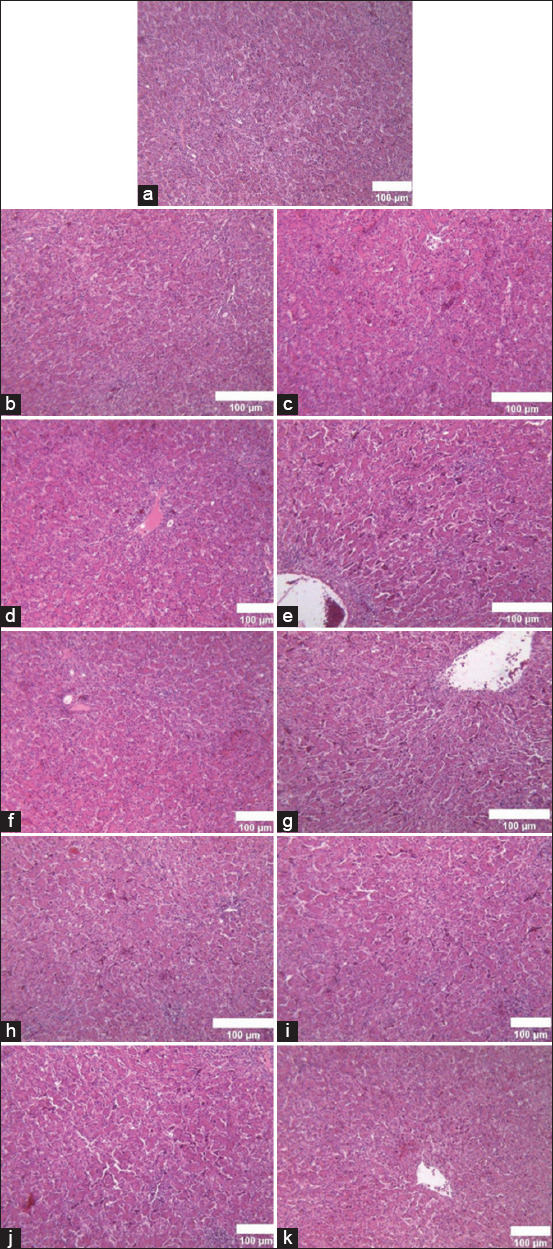
Comparison of liver histopathology using hematoxylin and eosin staining (200×). (a) Blank (b) DL-Met 70, (c) DL-Met 85, (d) DL-Met 100, (e) DL-Met 115,(f) DL-Met 135, (g) L-Met 70, (h) L-Met 85, (i) L-Met 100, (j) L-Met 115, (k) L-Met 135.

The liver histopathological examination revealed no significant changes or differences between the groups. All groups exhibited normal liver microstructure conditions with no notable variations in histopathological findings (Figure 4). Histopathological examination showed consistent findings without striking variations between groups (Figure 4).

## DISCUSSION

Extensive research has explored the comparative effects of L-Methionine and DL-methionine on liver metabolism and physiological processes in broiler chickens. These studies consistently highlight the distinct roles and metabolic efficiencies of the two forms of methionine [11, 12]. L-Methionine, a biologically active isomer, is directly involved in critical metabolic pathways, including protein synthesis, trans-sulfuration, and methylation. It plays a fundamental role in cell growth, tissue development, and overall health. In contrast, DL-Methionine, which is a racemic mixture of D- and L-isomers, requires the enzymatic conversion of the D-isomer to the active L-form, a process that could reduce its metabolic efficiency under certain physiological conditions [13]. These metabolic distinctions underline the necessity for precise formulations in poultry diets to optimize broiler growth and health outcomes [14].

The findings of this study reaffirm the superior efficacy of L-Methionine in improving broiler growth performance, particularly during the finisher stage when weight gain is most critical. This finding aligns with prior research demonstrating that L-Methionine supplementation positively affects growth performance, feed efficiency, and protein utilization [15]. As an essential amino acid, L-Methionine supports key physiological functions, including the synthesis of cysteine, glutathione, and other sulfur-containing compounds vital for cellular defense mechanisms and oxidative stress reduction. This biochemical role renders L-Methionine indispensable for broilers, particularly in diets that enhance growth while maintaining health without the use of growth-promoting antibiotics [16].

Supplementation with DL-Methionine, while somewhat less efficient in certain contexts, also offers notable benefits. Studies have shown that DL-Methionine contributes to improved feather development and carcass quality, making it a valuable component in poultry nutrition [17]. Its stability during feed processing and broad availability provides practical advantages, especially in large-scale feed formulations. Despite these benefits, DL-Methionine’s dependence on enzymatic conversion can lead to variability in metabolic utilization, depending on factors such as birds’ age, diet composition, and overall health status [17, 18].

The metabolic implications of methionine supplementation extend beyond growth performance. Reduced DAAO activity observed in this study suggests lower ammonia production in broilers supplemented with L-Methionine [19]. Excess ammonia can disrupt electrolyte balance, increase water intake, and lead to undesirable effects such as wetter feces and stronger ammonia odors in poultry housing. L-Methionine’s efficient metabolism minimizes these negative outcomes, making it a more environmentally friendly option for large-scale poultry production. This aspect is particularly relevant in the context of sustainable and eco-friendly poultry farming practices [20, 21].

L-Methionine’s direct involvement in trans-sulfuration and methylation pathways underscores its critical role in broiler metabolism. Differences in the use of L-Methionine and DL-Methionine may influence critical processes, including the synthesis of structural proteins and feather keratin [22]. These findings align with previous studies by Zhang *et al*. [23] that highlighted the importance of methionine in supporting optimal growth, feed conversion efficiency, and feather quality. The specific ratios of methionine to other amino acids, such as lysine, are also essential considerations in diet formulation because they directly impact broiler performance and nutrient use [24].

Histological analysis of liver tissue in this study revealed no significant pathological alterations across treatment groups. The microscopic structures of liver samples remained consistent with those of the control group, indicating that neither L-Methionine nor DL-Methionine supplementation caused any detectable hepatic abnormalities. This histological uniformity supports the safety of the two forms of methionine under tested dietary conditions. Moreover, the increased serum glutathione levels observed in the treatment groups highlighted the antioxidative benefits of methionine supplementation, further confirming its role in protecting cells against oxidative stress and maintaining liver health [25]. Elevated glutathione concentrations correlate with reduced oxidative damage and inflammation, emphasizing the overall health benefits of methionine-enriched diets [26].

While DL-Methionine remains a widely used and cost-effective option in commercial feed formulations, its metabolic limitations compared with L-Methionine highlight the need for tailored dietary strategies. DL-Methionine’s higher stability and ease of incorporation into feed matrices make it advantageous in certain contexts, especially when precise dosing and homogeneity are critical [27]. However, factors such as dosage, individual bird variability, and overall diet composition must be carefully managed to maximize the benefits of diet modification. Mixed findings in the literature further underscore the complexity of methionine metabolism and the importance of contextualizing results based on specific experimental conditions and objectives [6, 27].

Overall, the results of this study emphasize the multifaceted roles of L-Methionine and DL-Methionine in broiler nutrition. While both forms contribute to growth and metabolic health, L-Methionine demonstrates superior efficacy in terms of bioavailability, metabolic utilization, and impact on critical physiological pathways. Nevertheless, DL-Methionine remains a valuable component of broiler diets due to its practical advantages in feed formulation and its ability to improve growth performance [28, 29].

## CONCLUSION

This study demonstrated that L-Methionine was more effective in enhancing body weight gain, particularly in the finisher stage, compared to DL-Methionine, while DL-Methionine played a more significant role in liver metabolism by increasing DAAO activity and reducing oxidative stress, as indicated by lower serum glutathione concentrations. Histopathological analysis confirmed that neither form of methionine caused adverse hepatic alterations, supporting their safety in broiler diets. These findings highlight the distinct metabolic roles of DL-Methionine and L-Methionine, suggesting that L-Methionine is preferable for growth performance, while DL-Methionine may support liver function and antioxidant regulation.

This study provides comprehensive insights into the metabolic impact of methionine isomers, utilizing multiple physiological and biochemical parameters. The inclusion of varied dosage levels across three growth stages ensures a detailed assessment of methionine metabolism throughout broiler development. The controlled feeding environment, along with standardized diet formulations, enhances the internal validity of the findings. Despite its strengths, the study is limited by its short duration, covering only the standard broiler rearing period. It does not assess the long-term effects of methionine supplementation on reproductive performance or immunity. In addition, while liver metabolism markers were analyzed, gut microbiota composition and immune responses were not included, which could provide a more holistic understanding of methionine’s role in antibiotic-free poultry production.

Future research should focus on evaluating methionine metabolism beyond the broiler production phase, including post-slaughter meat quality, immune function, and gut microbiota interactions. Further studies should also explore optimal methionine inclusion levels in antibiotic-free diets to maximize both growth performance and metabolic health. Comparative studies on the cost-effectiveness of L-Methionine and DL-Methionine supplementation in large-scale poultry production would also be valuable. The findings of this study hold significant practical implications for commercial poultry nutrition. In antibiotic-free broiler production, L-Methionine can be prioritized for enhanced growth and feed efficiency, whereas DL-Methionine may be beneficial in supporting liver health and antioxidant balance. This differentiation can guide precision feeding strategies, allowing poultry producers to optimize methionine supplementation based on production goals, economic feasibility, and overall animal health. Given the growing global restrictions on AGPs, this study provides a viable nutritional alternative to support sustainable broiler production.

## AUTHORS’ CONTRIBUTIONS

AA and MS: Designed and conducted the study. TBTS, AAP, and AF provided materials and critical reviews. AF, HYP, and DAR conducted the literature search and prepared the manuscript. All authors have read and approved the final version of the manuscript.
